# 160 MoveWell: Providing Healthcare Professionals the opportunity to support patients with specific chronic conditions to be active for better health

**DOI:** 10.1093/eurpub/ckae114.018

**Published:** 2024-09-26

**Authors:** Caroline Kelleher

**Affiliations:** Cork Kerry Community Healthcare HSE, Cork, Ireland; LeisureWorld Bishopstown, Cork, Ireland; Cork Sports Partnership, Cork, Ireland

## Abstract

MoveWell is a programme dedicated to promoting physical activity among individuals with low activity levels and specific chronic diseases, aiming to reduce the risks associated with chronic conditions, and premature death. Despite the significant health benefits of physical activity, only 46% of the population meets national guidelines, with 64% expressing a desire for change but lacking guidance(1).

Healthcare professionals often feel uncertain about advising individuals with symptomatic medical conditions, and fear of exacerbating symptoms hinders people from increasing their physical activity(2). MoveWell supports the delivery of integrated secondary prevention services in the community that Healthcare professionals can be confident and happy referring to.

MoveWell focuses on providing suitable and confidence-inspiring activity opportunities for patients with chronic diseases, acknowledging their higher likelihood of relapse in physical activity behaviour.

**Description:**

This low-intensity resistance exercise class, led by qualified exercise professionals, caters to patients with Type II Diabetes, Asthma, COPD, and Cardiovascular Disease. MoveWell aims to enhance confidence for independent physical activity. It is not a rehabilitation programme but it does give Healthcare professionals the opportunity to recommend low-intensity activities in a safe environment.

**Development & Implementation:**

MoveWell was developed and implemented in partnership with local stakeholders. Primary care professional identified the need, Health Promotion and Improvement designed the solution. Cork Sports Partnership secured initial funding and LeisureWorld Bishopstown provide the venue and tutor.

Implementation began with one weekly class, expanding to two classes per week through targeted promotion to primary care staff and community outreach.

**Evaluation:**

MoveWell is still in a pilot stage and evaluation is just beginning.

The evaluation will involve participants completing a survey when they join the class, which asks about activity levels, confidence being physically active, and participation in community based activities. At 12 and 24 weeks they are surveyed again to monitor any changes.

**Dissemination & Conclusions:**

MoveWell’s expansion is underway, with plans to embed it in various networks, providing a cost-effective pathway that links chronic disease hubs, ICPOP, and local community networks. The programme’s structured approach, emphasising simplicity and accessibility, contributes to its potential to positively impact the physical activity levels of individuals with chronic conditions.

